# Characteristics of the lunar samples returned by the Chang’E-5 mission

**DOI:** 10.1093/nsr/nwab188

**Published:** 2021-10-14

**Authors:** Chunlai Li, Hao Hu, Meng-Fei Yang, Zhao-Yu Pei, Qin Zhou, Xin Ren, Bin Liu, Dawei Liu, Xingguo Zeng, Guangliang Zhang, Hongbo Zhang, Jianjun Liu, Qiong Wang, Xiangjin Deng, Caijin Xiao, Yonggang Yao, Dingshuai Xue, Wei Zuo, Yan Su, Weibin Wen, Ziyuan Ouyang

**Affiliations:** Key Laboratory of Lunar and Deep Space Exploration, National Astronomical Observatories, Chinese Academy of Sciences, Beijing 100101, China; Lunar Exploration and Space Engineering Center, Beijing 100190, China; Beijing Institute of Spacecraft System Engineering, Beijing 100094, China; Lunar Exploration and Space Engineering Center, Beijing 100190, China; Key Laboratory of Lunar and Deep Space Exploration, National Astronomical Observatories, Chinese Academy of Sciences, Beijing 100101, China; Key Laboratory of Lunar and Deep Space Exploration, National Astronomical Observatories, Chinese Academy of Sciences, Beijing 100101, China; Key Laboratory of Lunar and Deep Space Exploration, National Astronomical Observatories, Chinese Academy of Sciences, Beijing 100101, China; Key Laboratory of Lunar and Deep Space Exploration, National Astronomical Observatories, Chinese Academy of Sciences, Beijing 100101, China; Key Laboratory of Lunar and Deep Space Exploration, National Astronomical Observatories, Chinese Academy of Sciences, Beijing 100101, China; Key Laboratory of Lunar and Deep Space Exploration, National Astronomical Observatories, Chinese Academy of Sciences, Beijing 100101, China; Key Laboratory of Lunar and Deep Space Exploration, National Astronomical Observatories, Chinese Academy of Sciences, Beijing 100101, China; Key Laboratory of Lunar and Deep Space Exploration, National Astronomical Observatories, Chinese Academy of Sciences, Beijing 100101, China; Lunar Exploration and Space Engineering Center, Beijing 100190, China; Beijing Institute of Spacecraft System Engineering, Beijing 100094, China; Department of Nuclear Physics, China Institute of Atomic Energy, Beijing 102413, China; Department of Nuclear Physics, China Institute of Atomic Energy, Beijing 102413, China; State Key Laboratory of Lithospheric Evolution, Institute of Geology and Geophysics, Chinese Academy of Sciences, Beijing 100029, China; Key Laboratory of Lunar and Deep Space Exploration, National Astronomical Observatories, Chinese Academy of Sciences, Beijing 100101, China; Key Laboratory of Lunar and Deep Space Exploration, National Astronomical Observatories, Chinese Academy of Sciences, Beijing 100101, China; Key Laboratory of Lunar and Deep Space Exploration, National Astronomical Observatories, Chinese Academy of Sciences, Beijing 100101, China; Key Laboratory of Lunar and Deep Space Exploration, National Astronomical Observatories, Chinese Academy of Sciences, Beijing 100101, China; Institute of Geochemistry, Chinese Academy of Sciences, Guiyang 550081, China

**Keywords:** Chang’E-5, lunar soils, physical properties, petrography, mineralogy, chemistry

## Abstract

Forty-five years after the Apollo and Luna missions returned lunar samples, China's Chang’E-5 (CE-5) mission collected new samples from the mid-latitude region in the northeastern Oceanus Procellarum of the Moon. Our study shows that 95% of CE-5 lunar soil sizes are found to be within the range of 1.40–9.35 μm, while 95% of the soils by mass are within the size range of 4.84–432.27 μm. The bulk density, true density and specific surface area of CE-5 soils are 1.2387 g/cm^3^, 3.1952 g/cm^3^ and 0.56 m^2^/g, respectively. Fragments from the CE-5 regolith are classified into igneous clasts (mostly basalt), agglutinate and glass. A few breccias were also found. The minerals and compositions of CE-5 soils are consistent with mare basalts and can be classified as low-Ti/low-Al/low-K type with lower rare-earth-element contents than materials rich in potassium, rare earth element and phosphorus. CE-5 soils have high FeO and low Mg index, which could represent a new class of basalt.

## Teaser

The CE-5 sample is consistent with weathered mare basalts in mineralogy and petrochemistry, and is classified as low-Ti/low-Al/low-K type with lower REE (rare earth element) contents than KREEP (potassium, rare earth element, and phosphorus). This new sample characterized by high FeO and low Mg index could represent a new lunar basalt.

## INTRODUCTION

The Moon is the only natural satellite of the Earth and has always been an object of interest for scientists [1]. The first comprehensive lunar photographic atlas was completed by dozens of orbiter probes as early as the 1960s [2]. Based on these early images, the Moon is divided into two basic physiographic regions, namely, smooth maria and cratered highlands, both studded with craters of varying sizes. Studies of the lunar surface's morphology have indicated that the large craters originated from impact events and that the flat lunar maria might be filled with basalt [3,4]. Using the classical geological principle of superposition, the succession of events on the Moon was unraveled, a relative time scale was constructed and geological maps were prepared [5].

Samples are the key to promoting our scientific research, from remote observations to laboratory measurements. The returned lunar samples (∼382 kg [6,7]) from six Apollo and three Luna missions in the last century have significantly enhanced our understanding of the distribution, age and evolution of mare volcanism [8–12], the lunar mantle's composition and structure [13,14], the effect of physical properties on lunar exploration [15] and the Moon's surface processes (e.g. space weathering) [16]. The Apollo lunar samples were ‘the crown jewels of the scientific return of the Apollo missions’ [17]. However, Apollo lunar sampling had focused on areas non-representative of the most widespread lunar surface features [18]. These limited sample sites have restricted new cognition of the Moon.

**Figure 1.**
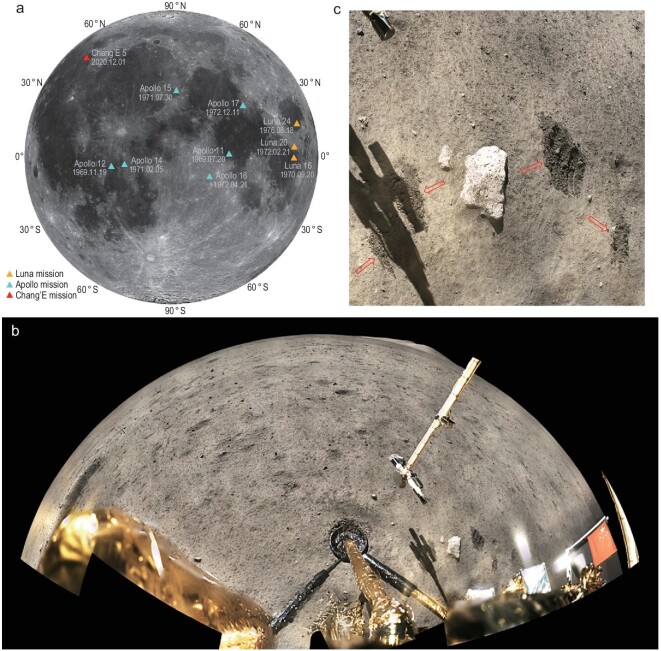
The distribution of lunar sampling sites and images of the CE-5 sampling site. (a) Lunar sampling sites and dates. Apollo and Luna sampling sites are within 30° of low latitude. The CE-5 sampling site is in a new area at mid-latitude. The image data are from the CE-1 global digital orthophoto map (DOM). Detailed information about these sites can be found in Supplementary Table 1. (b) The panoramic image of the CE-5 landing site. The 120 images taken by the panoramic camera onboard the CE-5 lander were mosaiced using fisheye projection, with a horizontal field of view ∼220°. (c) A partial image of CE-5 scooped sampling. The arrows show the trace of scooped sampling. All images are from the China Lunar Exploration Data Release Website (https://moon.bao.ac.cn).

Chang’E-5 (CE-5) is a sample return mission in China's lunar exploration strategy of ‘Orbit-Land-Sample return’. The sampling site is in the northeastern Oceanus Procellarum, with longitude and latitude of 51.916°W and 43.058°N. It is a new region with the highest sampling latitude to date, a latitude not reached by the previous Apollo and Luna sampling missions (Fig. 1). The returned CE-5 samples might carry information about the youngest volcanic activity on the Moon [19,20].

This study focuses on the preliminary examination of the lunar samples returned from the CE-5 mission, to obtain the physical properties, petrography, mineralogy and chemical characteristics of lunar soils and clasts, providing basic information for subsequent scientific research.

## RESULTS

### Geological context of the CE-5 lunar samples

Oceanus Procellarum, with the largest distribution of lunar mare basalts, is a prominent geochemically anomalous region on the lunar nearside. This region is enriched with thorium, uranium and potassium [21], with a relatively thin lunar crust [22], which might result in a potentially long history of volcanism [23] and a more complex thermal evolutionary history [24].

**Figure 2.**
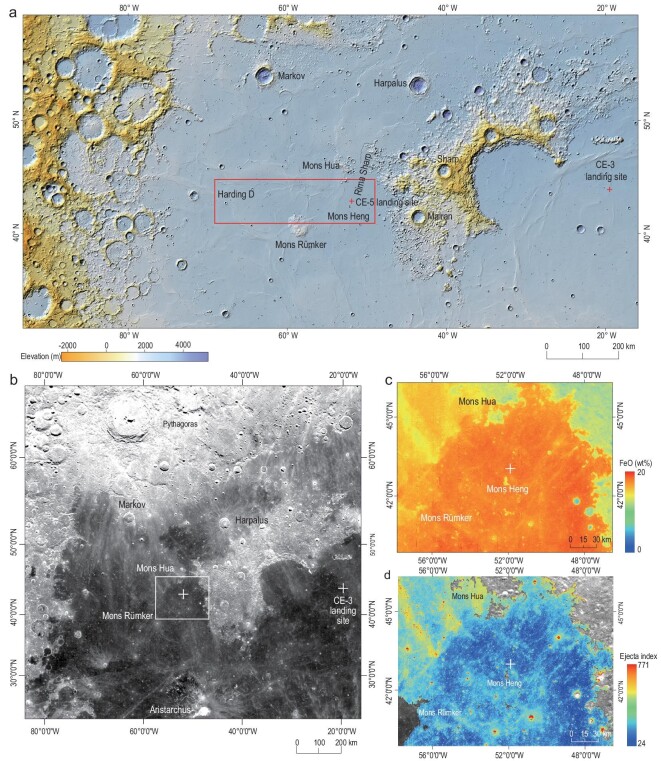
Geographical and geological backgrounds of the CE-5 lunar sample. The ‘+’ in all images are CE landing sites. (a) Topographic map with the red box showing the selected landing area. The topographic data are from the CE-2 global digital elevation model data. (b) Image and geological background of the landing site with the ejecta distribution. The white box is the area of c and d. Image data are from CE-2 global DOM. (c) Spectral concentration map of FeO (wt%) in the sampling area. It shows that the sampling site is uncontaminated by ejecta materials. FeO content is derived from https://astrogeology.usgs.gov/search/map/Moon/Kaguya/MI/MineralMaps/Lunar_Kaguya_MIMap_MineralDeconv_FeOWeightPercent_50N50S. (d) The enlarged map of the relative ejecta concentration index of the sampling area. The sampling site is free of ejecta contamination. All CE images are from the China Lunar Exploration Data Release Website (https://moon.bao.ac.cn).

The CE-5 landing area (41°–45°N, 69°–49°W) is in the relatively flat terrain of the mare plain in the northeastern Oceanus Procellarum (red box in Fig. 2a) [25]. The images of the sampling area show a relatively homogeneous texture and dark color (Figs 1b and 2b). Although the topography of the selected landing area is gentle, geometrically large terrains are distributed in the Oceanus Procellarum region (Fig. 2a and b), such as wrinkle ridges, Mons Rümker, craters of varied sizes and depths, and narrow lunar rilles. Most craters larger than 2 km in diameter are distributed in the western mare of the selected landing area, where the crater density is greater than in the eastern mare. Studies of crater size-frequency distribution indicate that the eastern mare of the selected landing area is the youngest geologic unit on the Moon [19,20]. Northeast of the sampling area is Rima Sharp, with an overall north–south orientation, a length of ∼566 km and a width of 0.8–3 km (Fig. 2a) [26,27].

CE-5 finally landed in the eastern part of the selected landing area on the mare surface to the northeast of Mons Heng and southeast of Crater Xu Guangqi (Fig. 2a, c and d). The sampling site’s surface is loose regolith scattered with different sized boulders (Fig. 1c).

The CE-5 selected landing area (Fig. 2a) is mostly distributed with ejecta rays that might originate from the Crater Pythagoras. Especially in the northwest of the selected landing area, the mare is covered with obvious north–northwest-oriented ejecta materials. However, these ejecta are cut off along the connecting line between Mons Hua and Mons Rümker (Fig. 2b) because the volcanic activity in the CE-5 sampling area might be younger than this impact event, resulting in lava flow covering and obliterating these former ejecta. Two sets of almost orthogonal ejecta (northwest and northeast), rather than Pythagoras ejecta, are distributed in the sampling area. The plume shape and faint concentration indicate the slight influence of these ejecta (Fig. 2c and d). Using FeO content as the tracing parameter, no obvious signs of ejecta can be observed within the sampling site (Fig. 2c). The ejecta index analysis shows that the sampling site is slightly contaminated compared to the darkest region of the selected landing area (Fig. 2d), consistent with Qian *et al*. [20], who proposed that the influence of the impact ejecta in the sampling area is less than 10%. Therefore, CE-5 lunar samples can be regarded as the product of weathered local rock with only minimal mixing of exotic ejecta materials.

### Physical properties of the CE-5 lunar samples

**Figure 3.**
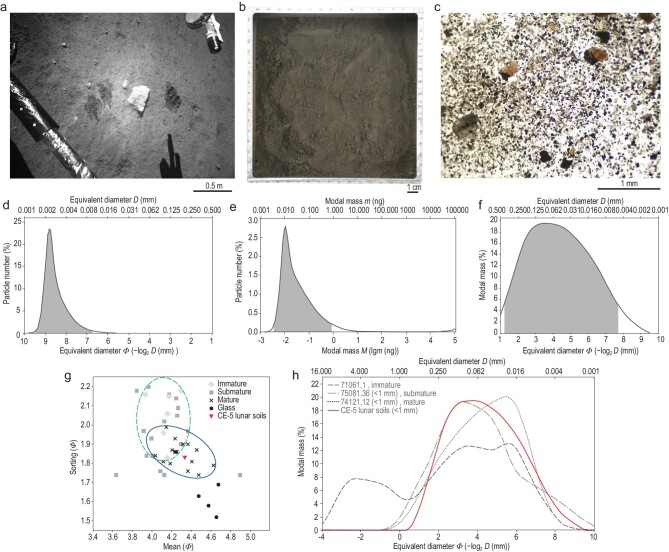
Image characteristics and particle size distribution of CE-5 lunar soils. (a) The surveillance camera image shows the characteristics of the lunar regolith at the sample site. The lunar regolith was mostly gray-black. The dark traces are the imprints after sampling. (b) A laboratory camera photo of scooped lunar regolith. (c) An image of lunar soil particles when magnified equivalent to the lunar soil grain size using a stereomicroscope (e.g. yellow-green olivine, white feldspar, brown-black pyroxene and brown glass). (d) The number (percent) distribution of particle size (equivalent diameter). Particle sizes range from 1.11 μm to 499.8 μm, with a mean of 3.96 μm, a median of 2.90 μm and a mode of 3.39 μm. Of the particles, 95% (the gray part) are distributed between 1.40 μm and 9.35 μm. (e) The modal mass (percent) distribution of particle size. The modal mass ranges from 0.0012 ng to 109177.8937 ng, with a mean of 0.5567 ng, a median of 0.0205 ng and a mode of 0.0095 ng. Of the particle mass, 95% (the gray part) is distributed between 0.0036 ng and 0.8304 ng. (f) The modal mass-grain size distribution of CE-5 lunar soils. Of the particle mass, 95% (the gray part) is distributed between 4.84 μm (Φ7.69) and 432.27 μm (Φ1.21), with a mean of 49.80 μm (Φ4.33), a mode of 88.38 μm (Φ3.50) and a median ($\mathit{\Phi}$_50_) of 52.54 μm (Φ4.25). (g) The grain size-sorting comparison between CE-5 and Apollo 17 lunar soils. CE-5 lunar soils tend to be mature. Apollo 17 lunar soil data are from Ref. [33]. (h) The comparison of modal mass-grain size distribution between CE-5 and Apollo lunar soils of varying maturity. The red solid line is CE-5 lunar soils, the dashed line is Apollo 17 (71061,1) immature lunar soils, the dot-dashed line is Apollo 17 (75081,36) submature lunar soils and the dotted line is Apollo 17 (74121,12) mature lunar soils. Apollo 17 lunar soil data are from Ref. [33].

The lunar regolith was mostly gray-black near the CE-5 sampling site (Fig. 3a). Although the lunar regolith appears gray-black (Fig. 3b), the minerals are colorful under the stereomicroscope (Fig. 3c). Refer to Supplementary Note 1 for details about sample preparation.

#### Particle size distribution of CE-5 lunar soils

We randomly selected 155 mg soils (No. CE5C0800YJFM001) from CE-5 scooped samples to systematically analyze its particle size distribution. The images of fully dispersed soil particles were taken using an optical microscope, followed by geometric measurements and statistical analysis. In total, 316 800 images of 2560 × 1920 pixels were acquired, and 299 869 867 particles of 1–500 μm (image resolution 0.4 μm) were identified. From the major axis, minor axis and projected area measurements, the shape parameters and modal mass of the lunar soil particles were calculated (Supplementary Table 2).

Our results show that 95% (in number) of CE-5 soil particle sizes (equivalent diameter) are distributed in the range of 1.40–9.35 μm (mean 3.96 μm, Fig. 3d), belonging to clay (<3.91 μm) to fine silt level (3.91–15.63 μm) [28]. Similarly, the grain mass of CE-5 samples is also concentrated. Of the lunar soil particles, 95% have a modal mass between 0.0036 ng and 0.8304 ng, with a mean of 0.5567 ng, a mode of 0.0095 ng and a median of 0.0205 ng (Fig. 3e). However, according to the particle size-mass distribution (Fig. 3f), 95% (in mass) of the CE-5 lunar soils is in the range of 4.84 μm (Φ7.69) to 432.27 μm (Φ1.21). The mean (($\mathit{\Phi}$_16_ + $\mathit{\Phi}$_50_ + $\mathit{\Phi}$_84_)/3), mode and median ($\mathit{\Phi}$_50_) particle sizes are 49.80 μm (Φ4.33), 88.38 μm (Φ3.50) and 52.54 μm (Φ4.25), respectively [29] (Fig. 3f). Therefore, the particle size of most lunar soils (in mass) is concentrated around 50 μm.

#### Density of CE-5 lunar soils

A Quantachrome ULTRAPYC 1200e analyzer was used to determine the true density of three lunar soil samples by helium displacement (one sample from CE5C0800YJFM005 and two samples from CE5C0100YJFM002). Each sample was measured nine times, and the average was taken as the true density of that soil sample. Results showed that the average natural bulk density of the three lunar soil samples was 1.2387 g/cm^3^, and the average true density was 3.1952 g/cm^3^, which is within the density range of terrestrial basalt.

#### Specific surface area of CE-5 lunar soils

We conducted 15 specific surface area (SSA) measurements on a 7.967 g soil sample (CE5C0100YJFM002) using a Quantachrome inert gas-adsorption SSA analyzer. Results showed that the SSA of the CE-5 whole soils is in the range of 0.55 m^2^/g to 0.57 m^2^/g, with an average of 0.56 m^2^/g.

A spherical particle's SSA is inversely proportional to its diameter and proportional to its total surface area. With a known SSA (measured value), the particle aggregate's mean size can be calculated if the particles are regular spheres (Supplementary Note 2). By comparing the calculated mean particle size with the measured value, the extent to which the particles within this particle aggregate deviate from the sphere can be inferred. Based on the measured SSA and true density of the CE-5 soil sample, the equivalent diameter of the CE-5 soil particle was calculated to be ∼3.35 μm. It is slightly lower than the average equivalent diameter (3.96 μm) of CE-5 soil particles measured in this study. Therefore, the particle shape of CE-5 lunar soils is less regular than that of the sphere and can reach 84.6% of the sphere macroscopically. This particle size is more consistent with that of Earth clay. The SSA of Earth clay (10–800 m^2^/g) [30] is much larger than that of the CE-5 lunar soil sample, indicating that CE-5 lunar soil particles are more regular or have a higher roundness than common Earth clay. However, we cannot rule out that the large porosity of Earth clay might contribute to its large surface area.

### Petrographic characteristics of CE-5 lunar samples

The particle sizes of CE-5 lunar samples are mostly distributed in the micron scale, and few rock fragments are larger than 1 cm. More than 95 wt% (in mass) of the CE-5 particles (equivalently ≥5 μm in size) in three polished sections were counted and analyzed by backscattered electron (BSE) images. The statistical results show that the average percentages for a single mineral, dual minerals and three or more minerals are 27.0%, 21.5% and 51.5%, and the average model masses are 57.4%, 32.1% and 10.5% (Supplementary Fig. 1), respectively. The rock clasts percentage (∼50%) in CE-5 lunar soils is high; however, the mass percentage is extremely low (∼10%), indicating that the volume/area of rock clasts is much smaller than that of single mineral clasts. This might be related to the coarse-grained structure of the original bedrock breaking and separating easily into single minerals during weathering.

During the sample separation process, many small fragments from 1 mm to 1 cm were collected. Through preliminary observations using a stereomicroscope and a scanning electron microscope at the National Astronomical Observatories, Chinese Academy of Sciences (NAOC), these fragments could be classified into basaltic clasts, agglutinates, breccias and glass.

#### Basaltic clasts

Basalt is the dominant and most significant lithic clast in the CE-5 lunar sample (many complex mineral grains smaller than 1 mm are of this type). It mostly comprises pyroxene, feldspar, olivine and ilmenite, with minor amounts of troilite, K-feldspar, quartz, tranquillityite, apatite, merrillite, baddeleyite and zirconolite. From detailed petrographic observations, five distinct textural types of basaltic clasts have been recognized.

**Figure 4.**
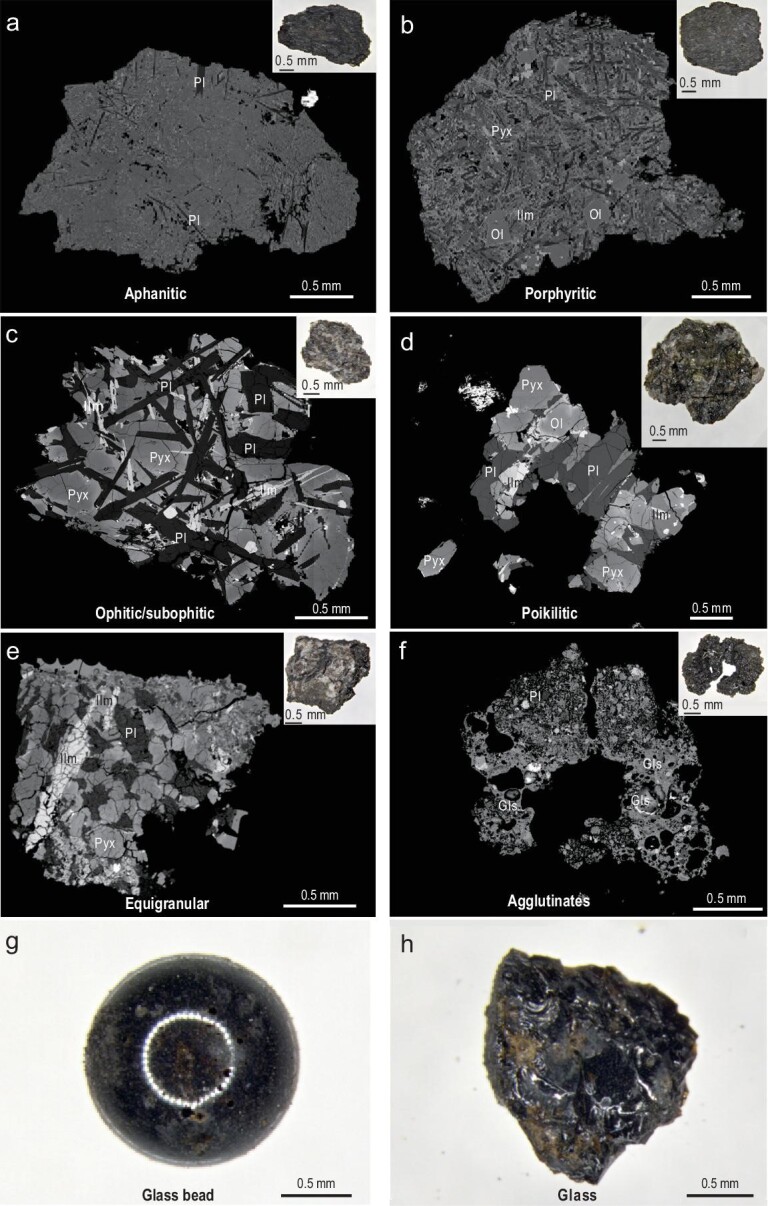
BSE images and stereomicrographs of typical basaltic clasts, agglutinate and glasses from the CE-5 lunar sample. (a)–(f) BSE images for basaltic clasts and agglutinate with different textures. The upper right corner of the BSE image is the stereomicrograph corresponding to each clast. (g), (h) Stereomicrographs of glass. Abbreviations: Pyx, pyroxene; Pl, plagioclase; Ol, olivine; Ilm, ilmenite; Glass, Gls.

**Aphanitic texture:** The mineral grains are extremely tiny (typically <0.01 mm), with fibrous intergrowth of plagioclase and ilmenite microcrystals oriented in the glass matrix (Fig. 4a).

**Porphyritic texture:** The mineral grain size is typically <0.05 mm. Plagioclase and ilmenite are stripe-like oriented. Olivine occurs as phenocrysts with grain sizes up to 0.5 mm (Fig. 4b).

**Ophitic/subophitic texture:** The grain size is fine, typically <0.1 mm. Euhedral plagioclase laths are filled with pyroxene and olivine grains (Fig. 4c).

**Poikilitic texture:** The mineral grains are coarse (0.1–0.5 mm). These coexisting silicate minerals, including pyroxene, olivine and plagioclase, show complex petrographic relationships (Fig. 4d).

**Equigranular texture:** The grain size ranges from 0.1 mm to 0.5 mm. The primary minerals of pyroxene and feldspar are approximately equal in size and have simple coexisting relationships (Fig. 4e).

The primary minerals in 29 basaltic clasts from seven polished sections were analyzed for their chemical composition (Supplementary Note 3 and Supplementary Table 3). The results showed that An (100 × Ca/(Ca + Na + K) molar ratio) of feldspar in these basaltic clasts is in the range of 75.0 to 95.5 (*n* = 172), with most being bytownite (average composition An_83.9_Ab_15.2_Or_0.9_, *n* = 166) (Supplementary Fig. 2a). Pyroxene is predominantly augite with an average composition of Wo_32.9_En_28.2_Fs_38.9_ (*n* = 90). Pigeonite is rare, with an average composition of Wo_17.8_En_14.4_Fs_67.8_ (*n* = 2) (Supplementary Fig. 2b). The Fe/Mn values for pyroxene in basaltic clasts range from 48.4 to 79.4, with an average of 61.6 (*n* = 92). The Fo (100 × Mg/(Mg + Fe) molar ratio) of olivine varies from 1.0 to 58.3, with an average of 37.2 (*n* = 73). Most olivines have Fo <50 (*n* = 54) (Supplementary Fig. 2c). The mineral compositions of these basaltic clasts correlate well with that of CE-5 whole soils, indicating that the lunar soils from the CE-5 landing site mostly comprises basalt weathered from the local basaltic bedrock.

#### Agglutinates

Agglutinates comprise lithic and mineral fragments welded together by the glass produced by melting due to small meteoroid impacts. Most agglutinates are irregular in shape, loose and easily broken, with relatively well-developed pores (Fig. 4f).

#### Breccias

The composition of breccias is complex, including mineral fragments and lithic clasts. The mineral fragments mostly comprise plagioclase, pyroxene, olivine and ilmenite, whereas the lithic clasts are almost exclusively basalt. The matrix mostly comprises plagioclase, pyroxene, olivine and glass, reflecting that this breccia is a bonding product of impacted basalt. The surface is occasionally covered with glass with highly variable content.

#### Glasses

According to morphological differences, glassy material in lunar soils can be divided into two principal categories. One is round glass beads (Fig. 4g), highly variable in color, mostly black and brown, with occasional green glass beads. The other is irregularly shaped glass fragments with obvious shell-like fractures (Fig. 4h). Brown pits are sometimes visible on the glass surface.

### Mineralogy of CE-5 lunar samples

#### Mineral species and abundance

**Figure 5.**
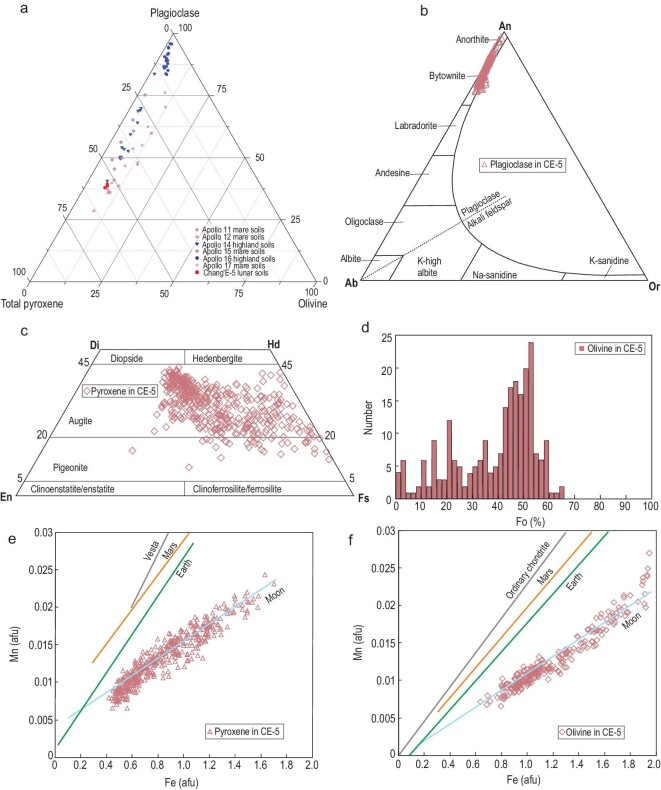
Mineral composition of CE-5 lunar soils compared with Apollo and Luna samples. (a) The triangular plot of major mineral abundances. CE-5 lunar soils are significantly enriched in pyroxene and low in olivine. The data of Apollo and Luna soils are from Refs [40,41]. (b)–(d) The mineral composition of plagioclase, pyroxene and olivine in CE-5 lunar soils. Plagioclase is mostly within the composition of bytownite. Pyroxene lies within the range of high-calcium pyroxene and is dominated by augite with a small amount of pigeonite. Olivine is mostly fayalite. (e), (f) Mn versus Fe atoms per formula unit in pyroxene and olivine in CE-5 lunar soils. Planetary trend lines are from Ref. [32] and references therein. Abbreviations: afu, atoms per formula unit.

The phase types and their contents of CE-5 lunar soils (∼100 mg, CE5C0800YJFM001-1, CE5C0100YJFM002-1 and CE5C0100YJFM002-2) were analyzed using a Bruker D8 Advance X-ray diffraction (XRD) analyzer and the Rietveld whole-pattern fitting method (Supplementary Note 4). The phase types identified by XRD and involved in Rietveld's whole-pattern fitting include augite, pigeonite, plagioclase, forsterite, fayalite, ilmenite, quartz, apatite and glass (Supplementary Table 4 and Supplementary Fig. 3). Results show that the content of plagioclase and augite in CE-5 lunar soils can reach ∼30% (Supplementary Table 5). The contents of pigeonite and glass are 10%–20%, and the other minerals are <10%. Olivine (mostly fayalite) is only 5%–6%, and ilmenite is 4%–5%. A small amount of apatite is present (up to 1.4%). However, no orthopyroxene was found in CE-5 soils. These features are consistent with the results of basaltic clast mineralogy, indicating that CE-5 lunar soils is equivalent to iron and calcium-rich basalt (Fig. 5a and Supplementary Table 5).

#### Mineral composition

Three major silicate minerals (monomineral fragments and minerals in lithic clasts) in 18 lunar soil polished sections were analyzed using an electron probe microanalyzer (EPMA). Two sections were taken from scooped sample bottles 01 to 07, three sections from scooped sample bottle 08, and one from scooped sample bottle 09 (Supplementary Note 3 and Supplementary Table 3).

The feldspar composition of CE-5 lunar soils is heterogeneous, with An varying from 76.1 to 97.6 (*n* = 277). More than 90% of the feldspar are bytownite (Fig. 5b), with an average composition of An_84.5_Ab_14.6_Or_0.9_ (*n* = 252). The content of anorthite is <10%, with an average composition of An_92.5_Ab_7.3_Or_0.2_ (*n* = 25). This feldspar composition is comparable to the Apollo basalt (An varies from 80.5 to 95.7) [31]. However, minor feldspar with An larger than 95.7 (*n* = 5) exists.

The pyroxene composition of CE-5 lunar soils is variable, mostly comprising augite followed by pigeonite and without orthopyroxene (Fig. 5c), correlating well with previous XRD analyses. The 425 analyzed data points of pyroxene indicate that augite accounts for 90% of pyroxene, with an average composition of Wo_31.4_En_26.3_Fs_42.3_ (*n* = 387), and pigeonite accounts for the remaining 10%, with an average composition of Wo_16.6_En_19.0_Fs_64.2_ (*n* = 38). The composition of pyroxene is also consistent with that of the Apollo basalts (Wo: 4.0–47.4; En: 0.4–67.8; Fs: 14.5–85.8) [31].

The olivine composition is variable among different grains, with Fo distributed in the range of 0.1 to 65.1 (*n* = 232) (Fig. 5d). Most olivine grains have Fo concentrated between 40 and 60, and 70% grains (*n* = 162) are Fe-rich (Fo values <50). In some cases, the olivine composition varies slightly from the core to the rim.

Mafic minerals (pyroxene and olivine) from different parent bodies (e.g. Earth, Moon and Mars) have different Fe/Mn atomic molar ratios due to the relative volatilities of Fe and Mn and the oxidation conditions of parent bodies. The pyroxene Fe/Mn ratio in CE-5 lunar soils ranges from 45 to 86.6, with an average of 62.6 (*n* = 425). The olivine MnO content is 0.28–0.94 wt%, and the Fe/Mn ratio is 72.1–121.5, with an average of 95.3 (*n* = 232). The Fe/Mn ratios for pyroxene and olivine are within the lunar trend line (Fig. 5e and f) and possess a genetic linkage with lunar environments, unlike Earth, Mars, asteroids and chondrites [32].

### Chemistry of CE-5 lunar samples

The bulk chemical composition of CE-5 lunar soils was analyzed using instrumental neutron activation analysis (INAA) and X-ray fluorescence spectrometer (XRF) (Supplementary Notes 4 and 5). CE5C0800YJFM002 and CE5C0800YJFM003 were analyzed using INAA (Supplementary Table 6), and CE5C0800YJFM002 was further analyzed using XRF (Table 1 and Supplementary Table 7). Most analyzed major elements (Na, Mg, Al, K, Ca, Ti, Fe and Mn) correlate well. The overall abundance of rare-earth element (REE) for CE-5 lunar soils correlates with that of Apollo 12 and is higher than most other lunar soils, except for Apollo 14 soils. REE patterns show higher light REE (LREE) concentrations, a negative Eu anomaly and lower heavy REE (HREE) concentrations.

**Table 1.** Bulk chemical composition for CE-5 lunar soils.

**XRF**															
**Element**	**SiO_2_**	**TiO_2_**	**Al_2_O_3_**	**FeO**	**MnO**	**MgO**	**CaO**	**Na_2_O**	**K_2_O**	**P_2_O_5_**	**Total**	**Mg#**			
wt%	42.2	5.00	10.8	22.5	0.28	6.48	11.0	0.26	0.19	0.23	**98.94**	**33.9**			
Uncertainty(*k* = 2)	0.34	0.06	0.18	0.33	0.03	0.35	0.10	0.210	0.15	0.05					
**INAA**															
**Element**	**Na**	**Mg**	**Al**	**K**	**Ca**	**Sc**	**Ti**	**V**	**Cr**	**Mn**	**Fe**	**Co**	**Ni**	**Zn**	**Rb**
ppm	3420	38600	57300	1510	74500	66	31100	95.8	1410	2150	174000	40	136	16.2	7.47
Uncertainty(*k* = 2)	205	2470	2600	151	4800	2.6	1600	8	56.4	86	7000	1.6	11	3.2	1.49
**Element**	**Zr**	**La**	**Cs**	**Ce**	**Pr**	**Sm**	**Eu**	**Gd**	**Tb**	**Dy**	**Ho**	**Lu**	**Ta**	**Th**	**U**
ppm	458	36.1	0.169	92.8	12.5	16.1	2.56	18.9	3.51	20.9	4.50	1.41	1.77	4.72	1.41
Uncertainty(*k* = 2)	34	1.4	0.038	3.7	2.22	0.6	0.1	0.77	0.28	1.4	1.4	0.08	0.18	0.28	0.28

## DISCUSSION

### Comparison of physical properties between CE-5 and Apollo samples

Particle size distribution is a fundamental physical parameter of lunar soils, affecting strength, compressibility, optical properties and thermal properties. During the lunar surface's weathering process, the soils will develop from immature, submature to mature as the surface exposure time increases. This process gradually decreases coarse particles and increases fine particles and agglutinates. Particle size analysis shows that about half of the immature Apollo lunar soils have a bimodal feature in their particle size distribution [33]. In contrast, most submature and mature Apollo lunar soils showed a single peak. The peak width narrows, exhibiting better sorting characteristics. The number and modal mass distributions of CE-5 lunar soil particles (Fig. 3d and e) have obvious single peaks, indicating their higher maturity. This implies that the CE-5 lunar soils are characterized by a relatively homogeneous origin, possibly from the continuous basaltic bedrock weathering. About 60% of the Apollo and Luna samples (47 of 80) have a larger mean size, and 88% (70 of 80) have a larger sorting (($\mathit{\Phi}$_84_ − $\mathit{\Phi}$_16_)/4 + ($\mathit{\Phi}$_95_ − $\mathit{\Phi}$_5_)/6.6) [34,35] than CE-5 soils (Supplementary Table 8). Therefore, CE-5 lunar soil samples are finer, better sorted (smaller sorting value) and relatively more mature than most Apollo and Luna soils (Fig. 3g). CE-5 lunar soil samples are different from the immature sample 71061,1 but similar to samples 75018,36 and 74121,12 (Fig. 3h), and can be classified as mature lunar soils.

The lunar soil density helps us understand its material composition, elasticity, thermal diffusivity, porosity and compressibility [36]. The specific gravity of CE-5 lunar soil samples is within the range of Apollo samples (2.9–3.24), but this value is significantly higher than that of Apollo 12 (12029, 12057) and Apollo 14 (14163, 14259) lunar soils (∼2.9). It is close to Apollo 11 (10004, 10005) and Apollo 15 lunar soils (15061: 3.24 g/cm^3^), but slightly lower than lunar basalt (10020: 3.25 g/cm^3^; 70017: 3.57 g/cm^3^; 70215: 3.44 g/cm^3^) [15]. CE-5 whole soils could comprise a mafic component, which is close to basalt.

The SSA describes the total surface area per unit mass of a collection of solid particles, reflecting the particle size in the collection and the irregularity degree of the particle shape. It is related to the adsorption properties and surface activity of the particle. On the lunar surface, micrometeorite impact, solar wind ion bombardment, and thermal expansion and contraction can fine, destroy, smooth, aggregate, or alter the size and texture of the grains composing lunar soils [35]. By measuring the SSA of lunar soil samples, one can understand the comprehensive effects of these lunar surface processes on lunar soil grains and their capacity to adsorb reactive molecules (e.g. water). The measured SSA of the Apollo samples ranges from 0.02 m^2^/g to 0.78 m^2^/g, with an average of 0.5 m^2^/g [15]. The SSA of the CE-5 sample is close to the average of the Apollo samples, especially close to 10084 (the mass-weighted average particle size of both samples is similar) [37]. The concentrated and small SSA values from Apollo to CE-5 lunar soils indicate relatively consistent particle size and surface properties of lunar soils globally. This demonstrates that gardening processes, such as micrometeorite bombardment, solar wind radiation, and thermal expansion and contraction are constant on the lunar surface [38]. Moreover, it is challenging for water to be stored either in Apollo or CE-5 lunar soil samples because of their small SSAs [39].

### CE-5 soils originated from weathered basalts

The total pyroxene content of the CE-5 lunar soils is ∼42%, significantly higher than that of Apollo lunar soils (0.9%–33.8%). Plagioclase content is ∼30.1%, slightly higher than that of Apollo mare samples (13.4%–20.0%), but significantly lower than that of Apollo 16 highland samples (28.1%–64.3%). The olivine content is ∼5.7%, close to that of Apollo lunar soils (0.3%–4.8%). The glass content is only 11.6%–20.0%, with an average of ∼16.6%, significantly lower than that of Apollo soils (25.4%–72.3%) [40,41]. CE-5 samples are mature soils according to the particle size results, and should have a high glass content. However, based on previous studies of Apollo samples, lunar soil maturity is not clearly related to high-intensity large meteorite impacts (producing impact glass) but to the injection of low-intensity micrometeorites, e.g. mm-size or smaller (producing agglutinitic glass). Thus, the low glass content of CE-5 samples indicates that they were less likely to be impacted by large meteorites, consistent with the lower crater density of the CE-5 landing area.

In the triangular plot of major mineral abundance, CE-5 samples are in the middle left of the map, similar to the Apollo 11, 12, 15 and 17 lunar mare samples (Fig. 5a). The highland lunar soils of Apollo 16 are mostly in the upper vertex in Fig. 5a, whereas Apollo 14 lunar highland soils exhibit a similar mineral distribution to mare soils due to the presence of ∼58% of Imbrium ejecta materials [42]. Compared with Apollo, CE-5 samples have higher pyroxene and lower plagioclase, and a typical mineral abundance of mare basalts rather than anorthosite and troctolite. Therefore, CE-5 lunar soils are mostly formed by accumulating weathered local basalt.

**Figure 6.**
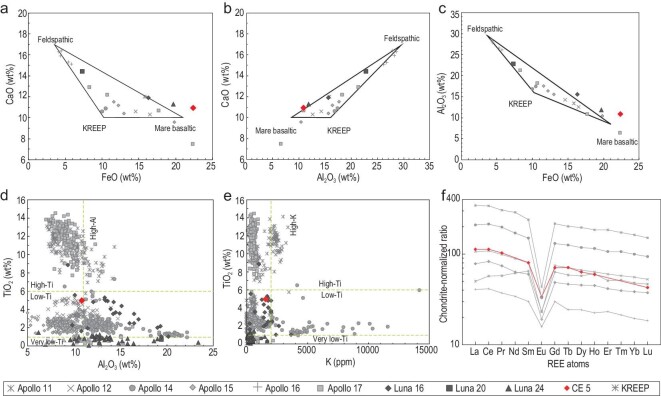
The chemical composition of CE-5 lunar soils compared with Apollo and Luna collections. (a)–(c) Elemental variations of Al_2_O_3_, CaO and FeO (database and triangles from Ref. [16]). (d), (e) TiO_2_, Al_2_O_3_ and K classification scheme of mare basalts. The protoliths of CE-5 lunar soils belong to the low-Ti/low-Al/low-K species (database from Ref. [4]). (f) Chondrite-normalized concentrations of REE in lunar soils as a function of an REE atom. The REE pattern of CE-5 lunar soils shows negative Eu anomalies. The database of Apollo samples is from Ref. [16] and the KREEP composition data are from Ref. [46]. Normalization values: 1.36C, where C represents the ‘Mean C1 Chondr.’ values of Table 1 in Ref. [48].

Since soils with few clasts larger than 1 cm dominate CE-5 lunar samples, it is challenging to perform a bulk chemical analysis of lunar rock. Compared with the Apollo and Luna missions, the CE-5 lunar soils are lower Al_2_O_3_ (10.8%) and CaO (11%) and higher FeO (22.5%), significantly different from feldspathic and KREEP (an acronym from the letters K (potassium), REE, and P (phosphorus)) endmembers and similar to the mare basaltic endmember (Fig. 6a–c). Therefore, the CE-5 lunar soils are clean and comprises *in situ* mare basalts. Combined with the results of the ejecta image analysis of the sampling area (Fig. 2), the CE-5 lunar soils are essentially free of contamination by exotic ejected materials. Therefore, we use the bulk chemical composition of lunar soils to represent the local basalt.

The SiO_2_ content of CE-5 lunar soils is as low as 42.2% but still within the range of mare basalts from Apollo missions (38%–48%). Compared with Earth's basalts, the SiO_2_ content of CE-5 lunar soils is significantly lower than that of subalkaline tholeiitic basalts and belongs to the ultramafic rock (SiO_2_ < 45%), whereas the MgO content of 6.5% is much lower than that of komatiites (MgO > 18%). According to the total alkali versus silica classification of the Earth's volcanic rocks, the basalt in the CE-5 landing site is in the region of picro-basalt based on SiO_2_ and alkali element (Na_2_O + K_2_O < 0.5%) compositions. However, their olivine content (5% in the XRD results presented previously) is much lower than that of Earth's picro-basalt (25%–40%). Therefore, the chemical composition of the protoliths forming CE-5 lunar soils is different from Earth basalts, and it is challenging to study CE-5 lunar soils using the classification criteria of Earth's basalt.

Mare basalts collected from Apollo and Luna missions are commonly defined as diverse types using TiO_2_, Al_2_O_3_ and K contents [4]. According to this classification scheme, CE-5 lunar soils belong to low-Ti/low-Al/low-K species (Fig. 6d and e), with significantly higher FeO content (22.5%, Fig. 6a and c) and a lower Mg index (Mg/(Mg + Fe) molar ratio = 33.9). Most mare basalts from Apollo and Luna collections have FeO contents below 22% and Mg# significantly higher than 35. Only the mare basalt from Luna 24 has a FeO content (22.4%) similar to the CE-5 lunar soils, but it still has a high Mg# [43].

The INAA results showed that the U, Th and K_2_O contents of CE-5 lunar soils are 1.41 ppm, 4.72 ppm and 0.19%, respectively, significantly lower than the U (4 ppm), Th (15.4 ppm) and K_2_O (0. 5%) contents of typical KREEP basalts [44,45]. Studies of Apollo samples have shown that the REE content of lunar KREEP composition is several hundreds to thousands of times higher than the chondrite CI-normalized ratio [46]. However, the REE content of CE-5 lunar soils is significantly lower than that of typical KREEP, indicating that the mare basalt in the CE-5 landing site is not a KREEP basalt (Fig. 6f). Although the REE content of CE-5 is significantly lower than KREEP, it is high among the mare basalts (La is ∼115 times higher than that of carbonaceous chondrites) [47] and close to the maximum REE content of mare basalts. In the REE pattern, CE-5 lunar soils are slightly enriched in LREE, with little fractionation between LREE and HREE. The overall REE pattern shape is similar to that of Apollo 12 lunar soils. There is a clear negative anomaly in Eu, a characteristic of mare basalt. This is also consistent with the expectation that the feldspathic lunar crust formed early in the lunar magma ocean model.

### Nature of mare basalts returned by the CE-5 mission

The particle size distribution and the similarity between the true density of CE-5 soils and Apollo basalt indicate a possible basaltic origin of the CE-5 sample. The most abundant minerals composing CE-5 soils are pyroxene, followed by plagioclase, with fewer amounts of ilmenite and olivine, indicating that basaltic composition dominates CE-5 soils. Specifically, pyroxene in CE-5 basalt is mostly augite with no orthopyroxene, and fayalite dominates olivine. The CE-5 lunar samples are low-Ti/low-Al/low-K basalt, exhibiting low SiO_2_ and alkaline (Na_2_O + K_2_O) content, moderate TiO_2_ and Al_2_O_3_, and very high FeO content. K, U, Th and REE contents of CE-5 soils are lower than KREEP materials but with a significant fractionation between different REE. Therefore, the CE-5 lunar sample could represent a new type of differentiated lunar basaltic rock.

## SUMMARY

The CE-5 mission returned the latest lunar samples after 45 years of sampling missions by the United States and the Soviet Union. The sampling site is far from the low latitudes of the Apollo Belt, with little disturbance from impact ejecta, and the samples possess properties of native basaltic bedrock.

The CE-5 lunar sample will open an epoch-making and unique window for studying lunar science in the following aspects: (i) the Moon's evolution; (ii) the timing, duration, volume, origin and emplacement mechanism of lunar volcanism in the northeastern Oceanus Procellarum; (iii) the bombardment history of the inner solar system; (iv) the galactic record in lunar regolith; (v) the lunar magnetic field and anomalies; and (vi) the relationship between lunar soil maturity and the contents of different glasses (impact and agglutinitic glass) [47].

## Supplementary Material

nwab188_Supplemental_File
