# Supplementary material for: Characteristics of the lunar samples returned by the Chang’E-5 mission
Source: Natl Sci Rev. 2021 Oct 14;9(2):nwab188. doi: 10.1093/nsr/nwab188 (PMC8974359; doi:10.1093/nsr/nwab188)
Supplement: nwab188_Supplemental_File [file nwab188_supplemental_file.docx]

**Supplementary Data**

**Characteristics of the lunar samples returned by Chang'E-5 mission**

Chunlai Li^1*^, Hao Hu^2*^, Meng-Fei Yang^3*^, Zhao-Yu Pei^2^, Qin Zhou^1^, Xin Ren^1^, Bin Liu^1^, Dawei Liu^1^, Xingguo Zeng^1^, Guangliang Zhang^1^, Hongbo Zhang^1^, Jianjun Liu^1^, Qiong Wang^2^, Xiangjin Deng^3^, Caijin Xiao^4^, Yonggang Yao^4^, Dingshuai Xue^5^, Wei Zuo^1^, Yan Su^1^, Weibin Wen^1^, Ziyuan Ouyang^1,6^.

1. Key Laboratory of Lunar and Deep Space Exploration, National Astronomical Observatories, Chinese Academy of Sciences, Beijing 100101, China

2. Lunar Exploration and Space Engineering Center, Beijing 100190, China

3. Beijing Institute of Spacecraft System Engineering, Beijing 100094, China

4. China Institute of Atomic Energy, Beijing 102413, China

5. State Key Laboratory of Lithospheric Evolution, Institute of Geology and Geophysics, Chinese Academy of Sciences, Beijing 100029, China

6. Institute of Geochemistry, Chinese Academy of Sciences, Guiyang 550081, China

Contents

[Supplementary Notes 3](#_Toc84085883)

[Supplementary Note 1. Preparation of the CE-5 lunar samples 3](#_Toc84085884)

[Supplementary Note 2. The equivalent diameter calculation based on Specific Surface Area measurement 3](#_Toc84085885)

[Supplementary Note 3. EPMA analysis of the polished sections for lunar soil and basaltic clasts 4](#_Toc84085886)

[Supplementary Note 4. XRD analysis of lunar soils 4](#_Toc84085887)

[Supplementary Note 5. INAA of lunar soil samples 5](#_Toc84085888)

[Supplementary Note 6. XRF analysis of luanr soil samples 5](#_Toc84085889)

[Supplementary tables 6](#_Toc84085890)

[Supplementary Table 1. Lunar samples returned from the Moon. 6](#_Toc84085891)

[Supplementary Table 2. The particle shape and size statistical results 6](#_Toc84085892)

[Supplementary Table 3. The number of EMPA analysis points for the polished sections of lunar soil and basalt clasts. 7](#_Toc84085893)

[Supplementary Table 4. Minerals used in the Rietveld whole-pattern fitting and the corresponding Powder Diffraction Files (PDF) of ICDD. 7](#_Toc84085894)

[Supplementary Table 5. Phases and abundances of CE-5 lunar soil samples. 8](#_Toc84085895)

[Supplementary Table 6. INAA scheme for lunar soil samples. 8](#_Toc84085896)

[Supplementary Table 7. XRF results for the sample of CE5C0800YJFM002. 9](#_Toc84085897)

[Supplementary Table 8. The particle size distribution parameters of the Apollo soil samples 9](#_Toc84085898)

[Supplementary Figures 12](#_Toc84085899)

[Supplementary Figure 1. Mineral assemblage of different particles from CE-5 lunar soil. 12](#_Toc84085900)

[Supplementary Figure 2. Composition of major silicate minerals in CE-5 basaltic clasts.. 13](#_Toc84085901)

[Supplementary Figure 3. The X-Ray diffraction patterns and Rietveld whole-pattern fitting results of the three lunar soil samples.. 14](#_Toc84085902)

[Supplementary References 15](#_Toc84085903)

# Supplementary Notes

## Supplementary Note 1. Preparation of the CE-5 lunar samples

On December 17, 2020, the lunar samples collected by the CE-5 mission were returned to Earth and stored in the NAOC. The total weight of CE-5 samples was ~1731 g. Using two sampling methods, they were divided into the scooped sample (prefix of sample number is CE5C) and drilled sample (prefix of sample number is CE5Z). The scooped sampling comprised 12 scoops from four sampling points at a sampling depth ~3 cm (Figs. 1c, 3a). In the laboratory, these scooped lunar samples were first transferred from the returned sample container using a special spoon into a 16 cm square stainless-steel container, in which the samples were thoroughly mixed and rock fragments larger than 1 mm were picked out. The sample surface was drawn into 16 squares, from which ⁓12 g of the sample was randomly scooped from 10 squares and placed into separate sample bottles (CE5C0100–CE5C1000). After one sampling process, the remaining samples were restirred thoroughly, redrawn into 16 squares, and rescooped randomly. This process was repeated 13 times until all samples were evenly divided into 10 sample bottles. This sampling process ensured a high degree of sample homogeneity among different bottles and allowed obtaining representative results even with a few samples. The drilled sample was packed in a 2.5 m long soft bag made of Kevlar. To maintain as much original location information as possible, the soft bag was cut into 11 sections using a special truncation tool, and each 15-cm long section was sampled and numbered at 1.5 cm intervals. Large rock clasts in the drilled sample were also picked out and numbered, and the residues that could not be removed from the soft bag were preserved as permanent storage samples. All unsealing, processing and sorting of lunar samples were conducted in a sealed, nitrogen-filled glove box. To preserve the original characteristics of the samples and avoid contamination from the Earth’s environment, water and oxygen in the glove box were controlled to be less than 1 ppm.

The scooped sample weighed ~1480 g and was highly mixed, overall gray-black and fine grain size (Fig. 3b). CE-5 lunar samples show obvious angularity under the microscope, indicating the weak effect of particle rounding. Although the lunar regolith appears gray-black, the minerals are colorful under the mineral grain size scale (e.g., yellow-green olivine, white feldspar, brown-black pyroxene, and brown glass) (Fig. 3c). The fine-grained fraction of lunar regolith with a particle size of less than 1 mm can be referred to as lunar soil, and most of the CE-5 regolith sample can be regarded as lunar soil in terms of particle size. CE-5 soil is highly adhesive, fine-grained, and often adheres together, making it challenging to distinguish lunar soil clumps from rock clasts with the naked eye. A layer of lunar soil is also typically attached to the surface of rock clasts, making it challenging to identify and select the fine-grained rock clasts. Residual samples after processing these samples are challenging to collect.

## Supplementary Note 2. The equivalent diameter calculation based on SSA measurement

The SSA of a spherical particle is inversely proportional to the diameter and can be calculated using the formula:

SSA= $\frac{6}{d*G*\rho_{w}}(m^{2}/g)$ (1)

Where *d* is the diameter of a sphere (equivalent diameter) in micrometers, *G* is the specific gravity, and $\rho_{w}$ is the density of water (1 g/cm^3^) [1]. The equivalent diameter was calculated to be ~3.35 μm based on the measured specific gravity (3.2) and SSA (0.56 $m^{2}/g$) of CE-5 lunar soils.

**Supplementary Note 3. EPMA analysis of the polished sections for lunar soil and basaltic clasts**

Major element compositions of plagioclase, pyroxene, and olivine were determined using an EPMA (JEOL JXA-8230) equipped with an energy disperse spectrometer (EDS, INCA) at NAOC. The minerals were analyzed at an accelerating voltage of 15 kV and an electron beam current of 10 nA with a spot diameter of 5 μm. The peak counting time was 20 s for each element, and the background time was 10 s. Natural minerals and synthetic glasses were used as standards, and the detection limits for most elements were 0.01 wt.%–0.03 wt.%. All data were corrected for atomic number (Z), X-ray absorption (A), and fluorescence (F) effects [2].

The analyzed polished sections include 18 lunar soil (Fig.5b–5d) and seven basalt clasts (Supplementary Figure 2 and Supplementary Table 3).

**Supplementary Note 4. XRD analysis of lunar soils**

Three lunar soils were prepared for XRD analysis. Sample 1 (CE5C0800YJFM001-1) is from CE5C0800YJFM001, and samples 2 (CE5C0100YJFM002-1) and 3 (CE5C0100YJFM002-2) are from CE5C0100YJFM002. The diffraction patterns of the three soil samples were collected using a Bruker D8 Advanced XRD instrument at NAOC. The identification and quantification of the mineral phases were analyzed using the Rietveld whole-pattern fitting method [3]. The three soil samples were first stirred thoroughly (at least for 1 h) to make the samples homogeneous. Large particles visible to the naked eye were removed to avoid preferred orientation. Then, the samples were scraped onto a flat surface using a quartz glass sheet and analyzed using XRD. After each measurement, the sample was restirred, rescraped, and reanalyzed using XRD. These XRD analyses were repeated 20 times for each sample. The data of 20 measurements were accumulated to further avoid preferred orientation. The measurement conditions were as following: the 2*θ* angles ranged from 5° to 90°, the increment was set to 0.015°, and the time for each step was 0.5 s. The whole pattern of each sample lasted one hour. Supplementary Figure 3 shows the three soil samples’ XRD patterns and Rietveld full-pattern fitting results by Jade software. The fitting errors (weighted residual error, Rwp) of the three samples were less than 7%, 6.06%, 5.04%, and 5.77%, respectively. The phases identified and involved in the whole-pattern Rietveld fitting included augite, pigeonite, plagioclase, forsterite, fayalite, ilmenite, quartz, and apatite. The standard diffraction patterns of each mineral phase are from the International Center for Diffraction Data, and the corresponding card number for each mineral phase is listed in Supplementary Table 4.

**Supplementary Note 5. INAA of lunar soil samples**

INAA of lunar soil was conducted at the China Institute of Atomic Energy. Lunar soil samples were irradiated in different reactors, including the miniature neutron source reactor (short irradiation) and swimming pool reactor (long irradiation). A γ-spectrometer system, including an HPGe detector (ORTEC, 60% efficiency, 1.85 keV resolution for 1332 keV of ^60^Co) and a multichannel analyzer (ORTEC DSPEC 50) was used for data acquisition and reduction. SPAN software was used for peak analysis and a hybrid k0-relative software ADVNAA for elemental concentration calculations. The specific experimental and data processing methods are detailed in [4–5]. Besides, the INAA scheme for lunar soil is listed in Supplementary Table 6.

Twenty-three elements in CE5C0800YJFM002 were quantified using long-irradiation neutron activation analysis, and seven elements in CE5C0800YJFM003 were quantified using short-irradiation neutron activation.

**Supplementary Note 6. XRF analysis of lunar soil samples**

The XRF analysis of CE-5 lunar soil samples was conducted using a wavelength dispersion (WD) XRF (WD-XRF) spectrometer (PANalytical AXIOS Minerals) at the Institute of Geology and Geophysics, Chinese Academy of Sciences. Of the sample, 30 mg lunar soil was used, and 3.0 g anhydrous powdered lithium borate (67% lithium tetraborate and 33% lithium metaborate mixed) was selected as the fluxing medium. The soil sample and the fluxing medium were mixed thoroughly in the Pt–Au crucible at a ratio of 1:100. Then, 0.15 ml of NH_4_Br aqueous solution (0.12 g/ml) was added as a release agent and fed into an M4 propane gas automatic fluxer (Claisse, Quebec, Canada). The soil sample was melted at high temperature in the M4 and cast into a disk-shaped glass sample. The prepared disc-shaped glass sample was measured three times using WD-XRF. Supplementary Table 7 shows the data results. The experimental and data processing methods are detailed in [6].

# Supplementary tables

## Supplementary Table 1. Lunar samples returned from the Moon^a^.

| **Mission** | **Landing time**  **(UTC)** | **Location** | **Destination** | **Returned sample**  **（kg）** |
| --- | --- | --- | --- | --- |
| Apollo 11 | 1969.7.20 | 0.67408°N, 23.47297°E | Mare Tranquillitatis | 21.55 |
| Apollo 12 | 1969.11.19 | 3.01239°S, 23.42157°W | Oceanus Procellarum | 34.30 |
| Apollo 14 | 1971.2.5 | 3.64530°S, 17.47136°W | Fra Mauro Highlands | 42.80 |
| Apollo 15 | 1971.7.30 | 26.13222°N, 3.63386°E | Hadley-Apennine | 76.70 |
| Apollo 16 | 1972.4.21 | 8.97301°S, 15.49812°E | Descartes Highlands | 95.20 |
| Apollo 17 | 1972.12.11 | 20.19080°N, 30.77168°E | Taurus-Littrow | 110.40 |
| Luna 16 | 1970.9.20 | 0.68°S, 56.30°E | Mare Fecunditatis | 0.101 |
| Luna 20 | 1972.2.21 | 3.57°N, 56.50°E | Apollonius Highlands | 0.030 |
| Luna 24 | 1976.8.18 | 12.75°N, 62.20°E | Mare Crisium | 0.1701 |
| CE-5 | 2020.12.1 | 43.058°N, 51.916°W | Statio Tianchuan, Northern Oceanus Procellarum | 1.731 |

**a.** Data of Apollo and Luna Missions were summarized from [7] and NASA website (<https://airandspace.si.edu/explore-and-learn/topics/apollo/apollo-program/landing-missions/sites.cfm> and https://nssdc.gsfc.nasa.gov/planetary/lunar/lunarussr.html); information of Chang’E-5 (CE-5) comes from this work.

## Supplementary Table 2. The particle shape and size statistical results (n=299,869,867)

| **Numbers** | **Statistical parameters** | **Minimum** | **Maximum** | **Median** | **Mode** | **Mean Size** | **Standard deviation** |
| --- | --- | --- | --- | --- | --- | --- | --- |
| 1 | Equivalent diameter^a^ (μm) | 1.11 | 499.80 | 2.90 | 2.21 | 3.96 | 3.39 |
| 2 | Aspect Ratio (AR)^b^ | 1.00 | 10.00 | 1.36 | 1.20 | 1.45 | 0.35 |
| 3 | Modal Mass^c^ (10^-6^mg) | 0.001 | 109177.894 | 0.02 | 0.01 | 0.56 | 52.61 |

**a.** The equivalent diameter (D) is defined as the diameter of a circle with the same projected area as the particle. It can be calculated as $D=2\sqrt{\frac{S}{\pi}}$. Here, S is the projected area (μm^2^), which is calculated by the total number of projected pixels occupied by lunar soil particles in the microscope image.

**b.** Aspect Ratio (AR) represents the characteristic of elongation or flattening of the particle. When AR equals to 1, the shape of the particle projection approximates a circle. The formula is $AR=\frac{D_{major}}{D_{minor}}$. The major axis ($D_{major}$) and minor axis ($D_{minor}$) are defined as those of the ellipse, which has the same area as the projected area of the soil particle.

**c.** Modal mass (M): The Modal mass is calculated using the Eq. M=k$\rho V$, where $\rho$ is the measured CE-5 lunar soil true density ($3.1952 g/cm^{3}$). V is the equivalent sphere volume and can be calculated by Eq.$V=\frac{4}{3}\pi\left( \frac{D}{2} \right)^{3}$. $k$ (0.5227) was derived from the deviation between the actual weighed mass and the calculated mass (m=$\rho V$) of 11 lunar soil samples (10mg~20mg each).

## Supplementary Table 3. The number of EMPA analysis points for the polished sections of lunar soil and basalt clasts.

| **Sample numbers** | **Plagioclase**  **（points）** | **Pyroxene**  **（points）** | **Olivine**  **（points）** |
| --- | --- | --- | --- |
| **Lunar soil polished sections** | | | |
| CE5C0100YJFM001GP | 21 | 22 | 10 |
| CE5C0100YJFM002GP | 17 | 14 | 6 |
| CE5C0200YJFM001GP | 14 | 18 | 10 |
| CE5C0200YJFM002GP | 18 | 26 | 12 |
| CE5C0300YJFM001GP | 16 | 32 | 11 |
| CE5C0300YJFM002GP | 15 | 14 | 11 |
| CE5C0400YJFM001GP | 15 | 31 | 27 |
| CE5C0400YJFM002GP | 9 | 33 | 19 |
| CE5C0500YJFM001GP | 9 | 16 | 8 |
| CE5C0500YJFM002GP | 12 | 29 | 15 |
| CE5C0600YJFM001GP | 13 | 39 | 22 |
| CE5C0600YJFM002GP | 13 | 21 | 12 |
| CE5C0700YJFM001GP | 19 | 27 | 12 |
| CE5C0700YJFM002GP | 15 | 26 | 19 |
| CE5C0800YJFM00102GP | 29 | 20 | 13 |
| CE5C0800YJFM00103GP | 11 | 7 | 3 |
| CE5C0800YJFM00104GP | 21 | 15 | 14 |
| CE5C0900YJFM001GP | 10 | 35 | 8 |
| **Basaltic clasts polished sections** | | | |
| CE5C0000YJYX001GP | 25 | 28 | 21 |
| CE5C0000YJYX03501GP | 26 | 13 | 10 |
| CE5C0000YJYX041GP | 15 | 22 | 5 |
| CE5C0000YJYX042GP | 19 | 20 | 17 |
| CE5C0000YJYX057GP | 22 | 1 | 2 |
| CE5C0000YJYX063GP | 43 | 5 | 6 |
| CE5C0000YJYX070GP | 22 | 3 | 12 |

## Supplementary Table 4. Minerals used in the Rietveld whole-pattern fitting and the corresponding Powder Diffraction Files (PDF) of ICDD.

|  | **Mineral** | **PDF numbers of ICDD** |
| --- | --- | --- |
|  | Plagioclase | PDF04-011-6816 |
|  | Augite | PDF98-000-0102 |
|  | Pigeonite | PDF01-076-2962 |
|  | Forsterite | PDF01-085-1207 |
|  | Fayalite | PDF04-008-8540 |
|  | Ilmenite | PDF04-007-2813 |
|  | Apatite | PDF01-080-7126 |
|  | Quartz | PDF98-000-0369 |

## Supplementary Table 5. Phases and abundances of CE-5 lunar soil samples.

| **Phase** | **Samples and abundances（wt.%）** | | | |
| --- | --- | --- | --- | --- |
|  | CE5C0800YJFM001-1 | CE5C0100YJFM002-1 | CE5C0100YJFM002-2 | mean |
| **Plagioclase** | 29.1 | 31.8 | 29.3 | 30.1 |
| **Augite** | 28.8 | 35.0 | 28.8 | 30.9 |
| **Pigeonite** | 13.3 | 9.1 | 10.9 | 11.1 |
| **Forsterite** | 2.0 | 1.5 | 1.4 | 1.6 |
| **Fayalite** | 3.5 | 4.9 | 4.0 | 4.1 |
| **Ilmenite** | 4.6 | 4.3 | 4.6 | 4.5 |
| **Apatite** | 0.1 | 1.4 | 0.7 | 0.7 |
| **Quartz** | 0.4 | 0.4 | 0.3 | 0.4 |
| **Glass** | 18.2 | 11.6 | 20.0 | 16.6 |

## Supplementary Table 6. INAA scheme for lunar soil samples.

| **Sample** | **Thermal neutron flux（φ_th_）** | **Ratio of thermal neutron to -epi thermal neutron（φ_th_/φ_e_）** | **Irradiation time（t_i_）** | **Decay time**  **（t_d_）** | **Measurement time（t_c_）** | **Elements** |
| --- | --- | --- | --- | --- | --- | --- |
| CE5C0800  YJFM002 | 3.9 x 10^13^  n·cm^-2^·s^-1^ | 24 | 24 hours | 4 days |  | K |
|  |  |  |  | 6-10 days |  | Na, Ho, La, Sm, Lu, U, Pr |
|  |  |  |  | 20 days |  | Cr, Fe, Sc, Co, Ni, Zr, Ce, Th, Ta, Tb, Eu, Gd, Rb |
|  |  |  |  | 25 days |  | Cs, Zn |
| CE5C0800  YJFM003 | 9 x 10^11^  n·cm^-2^·s^-1^ | 20 | 300 seconds | 150 seconds | 500 seconds | Al, Mg, Ti, Mn, Ca, V |
|  |  |  |  | 8 hours | 2000 seconds | Dy |

## Supplementary Table 7. XRF results for the sample of CE5C0800YJFM002 (wt.%).

|  | **Number 1** | **Number 2** | **Number 3** | **Mean** | **Uncertainty(k=2)^a^** |
| --- | --- | --- | --- | --- | --- |
| SiO_2_ | 42.1 | 42.2 | 42.3 | 42.2 | 0.34 |
| TiO_2_ | 5.00 | 5.00 | 5.00 | 5.00 | 0.06 |
| Al_2_O_3_ | 10.8 | 10.9 | 10.8 | 10.8 | 0.18 |
| TFeO | 22.5 | 22.5 | 22.5 | 22.5 | 0.33 |
| MnO | 0.28 | 0.28 | 0.28 | 0.28 | 0.03 |
| MgO | 6.49 | 6.47 | 6.48 | 6.48 | 0.35 |
| CaO | 11.0 | 10.9 | 11.0 | 11.0 | 0.10 |
| Na_2_O | 0.29 | 0.26 | 0.23 | 0.26 | 0.10 |
| K_2_O | 0.18 | 0.19 | 0.19 | 0.19 | 0.15 |
| P_2_O_5_ | 0.22 | 0.23 | 0.24 | 0.23 | 0.05 |
| Total | 98.86 | 98.93 | 99.02 | 98.94 |  |

**a.** The uncertainties of the mean value includes: 1, the uncertainty of sample preparation and measurement precision (measurement repeatability component assessment); 2, the uncertainty of curve fitting; 3, the uncertainty of all reference substances used to build the standard curve; 4, the uncertainty of weighing.

## Supplementary Table 8. The particle size distribution parameters of the Apollo soil samples (＜1mm)^a^

| **Sample Number** | **Maturity** | **Mean,Φ** | **Median,Φ** | **Mode,Φ** | **Sorting,Φ** |
| --- | --- | --- | --- | --- | --- |
| 12003,11 | submature | 3.93 | 3.87 | 3.50 | 2.03 |
| 12023,25 | mature | 4.39 | 4.22 | 4.50 | 2.46 |
| 12033,464 | immature | 4.21 | 4.12 | 4.50 | 2.53 |
| 12033,37 | immature | 3.78 | 4.04 | 4.50 | 2.12 |
| 12033,45 | immature | 4.28 | 4.07 | 3.50 | 2.57 |
| 12041,23 | mature | 4.53 | 4.33 | 4.50 | 2.43 |
| 12044,12 | submature | 4.47 | 4.15 | 3.50 | 2.25 |
| 12044,40 | submature | 3.54 | 3.46 | 2.50 | 1.74 |
| 12070,104 | submature | 4.30 | 4.07 | 3.50 | 2.25 |
| 12070,166 | submature | 4.26 | 4.32 | 4.50 | 2.30 |
| 14230,67 | submature | 3.89 | 4.26 | 4.50 | 1.89 |
| 14230,93 | submature | 3.73 | 4.02 | 4.50 | 1.80 |
| 14230,76 | submature | 3.90 | 4.07 | 4.50 | 2.36 |
| 14230,80 | submature | 3.30 | 3.46 | 4.50 | 1.94 |
| 14230,83 | submature | 2.94 | 2.98 | 4.50 | 2.06 |
| 15001,265 | immature | 3.81 | 3.78 | 3.50 | 2.13 |
| 15001,266 | immature | 4.29 | 4.30 | 4.50 | 2.13 |
| 15001,269 | submature | 4.02 | 4.15 | 4.50 | 2.17 |
| 15001,270 | submature | 4.22 | 4.25 | 4.50 | 2.17 |
| 15002,24 | submature | 3.81 | 4.00 | 4.50 | 2.02 |
| 15002,327 | submature | 3.78 | 4.06 | 4.50 | 2.43 |
| 15002,329 | submature | 3.72 | 3.96 | 4.50 | 2.52 |
| 15002,331 | submature | 4.39 | 4.46 | 4.50 | 2.13 |
| 15002,333 | submature | 4.32 | 4.24 | 4.50 | 2.20 |
| 15003,321 | immature | 4.14 | 4.22 | 4.50 | 2.41 |
| 15003,322 | immature | 3.25 | 3.19 | 1.50 | 1.98 |
| 15003,324 | submature | 4.41 | 4.44 | 4.50 | 2.05 |
| 15004,24 | submature | 3.88 | 3.83 | 3.50 | 1.82 |
| 15004,131 | submature | 4.26 | 4.29 | 4.50 | 2.03 |
| 15004,132 | submature | 4.24 | 4.24 | 4.50 | 2.14 |
| 15004,133 | immature | 4.30 | 4.36 | 4.50 | 2.31 |
| 15005,391 | submature | 4.45 | 4.41 | 4.50 | 1.92 |
| 15005,393 | submature | 4.39 | 4.50 | 5.50 | 2.15 |
| 15005,395 | submature | 4.48 | 4.54 | 4.50 | 1.94 |
| 15006,17 | submature | 4.07 | 4.07 | 3.50 | 1.79 |
| 15006,24 | submature | 3.97 | 4.14 | 5.50 | 1.73 |
| 15006,200 | mature | 4.20 | 4.18 | 4.50 | 1.91 |
| 15006,201 | submature | 4.23 | 4.24 | 4.50 | 1.83 |
| 15006,202 | mature | 4.33 | 4.33 | 4.50 | 2.05 |
| 15006,203 | mature | 3.97 | 4.06 | 4.50 | 2.23 |
| 15006,204 | mature | 4.25 | 4.25 | 4.50 | 2.07 |
| 15007,181 | submature | 2.86 | 2.78 | 2.50 | 1.77 |
| 15008,206 | submature | 4.67 | 4.37 | 4.50 | 2.36 |
| 15008,205 | submature | 4.66 | 4.38 | 4.50 | 2.35 |
| 15008,204 | mature | 4.64 | 4.35 | 4.50 | 2.43 |
| 15008,203 | submature | 4.65 | 4.38 | 4.50 | 2.45 |
| 15008,202 | submature | 4.70 | 4.42 | 4.50 | 2.41 |
| 15008,201 | mature | 4.51 | 4.21 | 4.50 | 2.50 |
| 60006,185 | mature | 3.35 | 3.05 | 2.50 | 1.81 |
| 60006,184 | mature | 3.94 | 3.72 | 3.50 | 2.28 |
| 60007,206 | mature | 3.93 | 3.90 | 5.50 | 2.18 |
| 60007,217 | mature | 3.96 | 3.90 | 3.50 | 2.23 |
| 60007,221 | mature | 3.94 | 3.98 | 5.50 | 2.12 |
| 60007,222 | mature | 4.08 | 4.15 | 5.50 | 2.09 |
| 60007,232 | mature | 4.15 | 4.20 | 5.50 | 1.95 |
| 60007,243 | mature | 4.07 | 4.15 | 5.50 | 2.11 |
| 60007,248 | mature | 3.81 | 3.94 | 5.50 | 2.21 |
| 66075,16 | immature | 5.39 | 5.16 | 4.50 | 2.48 |
| 70008,239 | immature | 4.17 | 4.15 | 3.50 | 2.27 |
| 70008,231 | immature | 3.71 | 3.72 | 3.50 | 2.57 |
| 70008,228 | immature | 3.99 | 3.93 | 3.50 | 2.13 |
| 70008,220 | immature | 3.42 | 3.41 | 3.50 | 2.07 |
| 72151,2 | mature | 4.26 | 4.31 | 4.50 | 2.03 |
| 74001,125 | immature | 4.69 | 4.42 | 4.50 | 2.20 |
| 74001,119 | immature | 4.69 | 4.30 | 4.50 | 2.19 |
| 74001,113 | immature | 4.47 | 4.18 | 3.50 | 2.09 |
| 74001,107 | immature | 4.73 | 4.45 | 4.50 | 2.01 |
| 74001,98 | immature | 4.82 | 4.44 | 4.50 | 2.09 |
| 74002,182 | immature | 4.83 | 4.62 | 4.50 | 1.79 |
| 74002,181 | immature | 5.10 | 4.67 | 4.50 | 2.07 |
| 74002,180 | immature | 4.60 | 4.61 | 5.50 | 1.66 |
| 74002,179 | immature | 5.03 | 4.71 | 4.50 | 2.21 |
| 74002,178 | immature | 4.96 | 4.58 | 4.50 | 2.21 |
| 74002,177 | immature | 5.06 | 4.70 | 4.50 | 2.27 |
| 74002,176 | immature | 5.16 | 4.78 | 4.50 | 2.25 |
| 74002,175 | immature | 5.14 | 4.74 | 4.50 | 2.18 |
| 79002,145 | submature | 4.73 | 4.38 | 3.50 | 2.50 |
| 79002,143 | mature | 4.52 | 4.24 | 4.50 | 2.36 |
| 79002,142 | mature | 4.48 | 4.22 | 3.50 | 2.50 |
| 79002,140 | mature | 4.41 | 4.15 | 3.50 | 2.39 |

**a.** The data is sourced from the reference [8] and the calculation of the particle size distribution parameters of Mean, Median, Mode and Sorting (Inclusive Graphic Standard Deviation), see the reference [9].

# Supplementary Figures


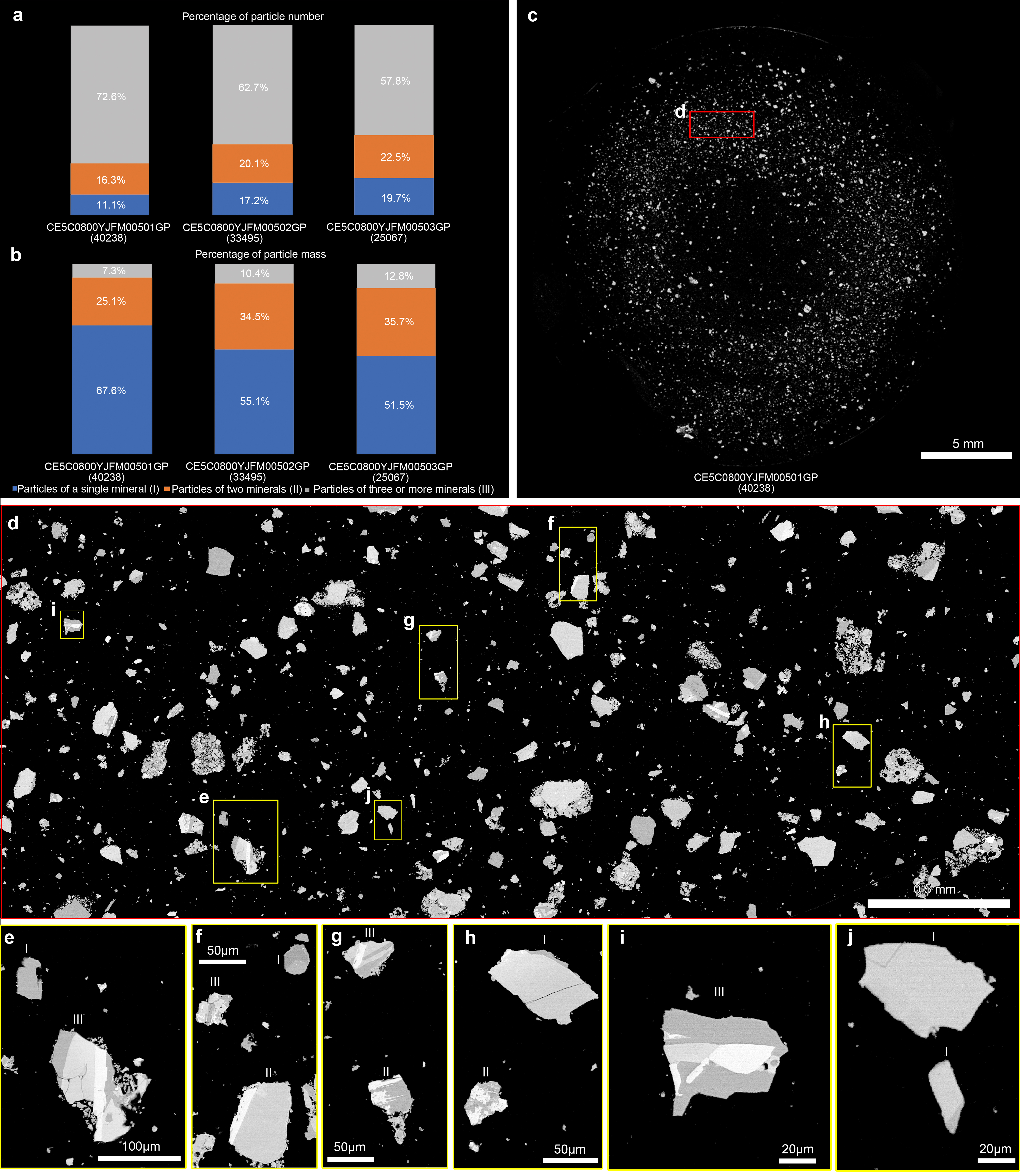


## Supplementary Figure 1. Mineral assemblage of different particles from CE-5 lunar soil. (a) Percentage of particle number for three different mineral assemblage in the polished sections of lunar soil. (b) Percentage of particle mass for three different mineral assemblage in the polished sections of lunar soil. (c) BSE image for the whole polished section of lunar soil. (d) Enlarged image of the area marked by the red square in Fig. 5c. (e) ~ (j) Typical BSE images for single mineral, dual minerals and three or more minerals. Abbreviations: I: single mineral; II: dual minerals; III: three and above minerals.


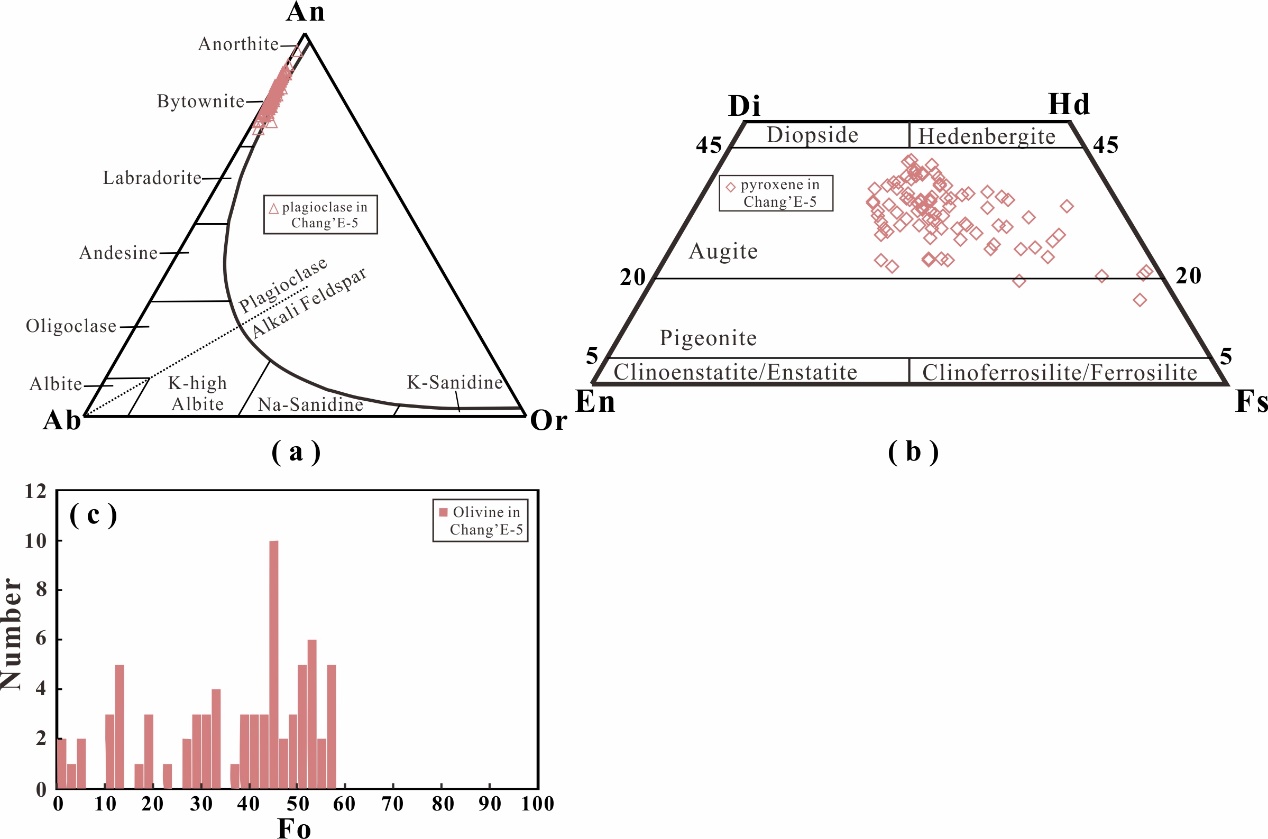


## Supplementary Figure 2. Composition of major silicate minerals in CE-5 basaltic clasts. Composition of plagioclase (a), pyroxenes (b) and olivine (c). All the data are from the seven basaltic clasts of CE-5.





## Supplementary Figure 3. The X-Ray diffraction patterns and Rietveld whole-pattern fitting results of the three lunar soil samples. Original patterns represent the XRD measured spectra of the samples, Rietveld Fitted Patterns represent the fitted spectra, Difference Patterns demonstrate the difference between the fitted spectra and the measured spectra (offset 45000 for clarity). The value on the difference pattern curve close to the straight line (45000), suggesting a good fitting result. Amorphous patterns are the diffraction spectra of the fitted glass. In addition, the Background are the fitted background curves of the original patterns (offset 50000 for clarity).

# Supplementary References

1. Carrier III WD, Olhoeft GR and Mendell W. Physical Properties of the Lunar Surface. In: Heiken GH, Vaniman DT, French BM (eds) Lunar source book—A User’s Guide to the Moon. Cambridge: Cambridge University Press, 1991.

2. Reed SJB. Electron microprobe analysis (2nd edition). Cambridge University Press, Cambridge, 1993.

3. Bish DL and Post JE, Quantitative mineralogical analysis using the Rietveld full-pattern fitting method. Amer Min 1993; 78: 932-940.

4. Tian WZ, Ni BF and Wang PS et al., Metrological role of neutron activation analysis. IA. Inherent characteristics of relative INAA as a primary ratio method of measurement. Accred Qual Assur 2001; 6: 488-492.

5. Tian WZ, Ni BF and Wang PS et al., Metrological role of neutron activation analysis. IB. Inherent characteristics of relative INAA as a primary ratio method of measurement. Accred Qual Assur 2002; 7: 7-12.

6. Xue DS, Su BX and Zhang DP et al., Quantitative verification of 1:100 diluted fused glass beads for X-ray fluorescence analysis of geological specimens. Journal of Analytical Atomic Spectrometry, 2020; 35: 2826–2833.

7. McCubbin FM, Herd CDK and Yada T et al. Advanced Curation of Astromaterials for Planetary Science. Space Sci Rev 2019; 215: 48.

8. Graf JC, Lunar soils grain size catalog. National Aeronautics and Space Administration, Office of Management, Scientific and Technical Information Program, 1993; 1265.

9. Folk RL. Petrology of Sedimentary Rocks. Hemphill’s, Austin, 1968.
